# What Africa can do to accelerate and sustain progress against malaria

**DOI:** 10.1371/journal.pgph.0000262

**Published:** 2022-06-24

**Authors:** Fredros Okumu, Margaret Gyapong, Núria Casamitjana, Marcia C. Castro, Maurice A. Itoe, Friday Okonofua, Marcel Tanner

**Affiliations:** 1 Ifakara Health Institute, Ifakara, Tanzania; 2 Centre for Health Policy and Implementation Research, Institute of Health Research, University of Health and Allied Sciences, Ho, Ghana; 3 Barcelona Institute for Global Health (ISGlobal), Hospital Clinic–University of Barcelona, Barcelona, Spain; 4 Department of Global Health and Population, Harvard T.H. Chan School of Public Health, Boston, Massachusetts, United States of America; 5 Department of Immunology and Infectious Diseases, Harvard T.H. Chan School of Public Health, Boston, Massachusetts, United States of America; 6 Department of Obstetrics and Gynaecology, School of Medicine, University of Benin, Benin City, Nigeria; 7 Department of Medical Parasitology and Infection Biology, Swiss Tropical and Public Health Institute, Basel, Switzerland; 8 University of Basel, Basel, Switzerland; Menzies School of Health Research, AUSTRALIA

## Abstract

After a longstanding global presence, malaria is now largely non-existent or suppressed in most parts of the world. Today, cases and deaths are primarily concentrated in sub-Saharan Africa. According to many experts, this persistence on the African continent reflects factors such as resistance to insecticides and drugs as well as insufficient access to essential commodities such as insecticide-treated nets and effective drugs. Crucially, however, this narrative ignores many central weaknesses in the fight against malaria and instead reinforces a narrow, commodity-driven vision of disease control. This paper therefore describes the core challenges hindering malaria programs in Africa and highlights key opportunities to rethink current strategies for sustainable control and elimination. The epidemiology of malaria in Africa presents far greater challenges than elsewhere and requires context-specific initiatives tailored to national and sub-national targets. To sustain progress, African countries must systematically address key weaknesses in its health systems, improve the quality and use of data for surveillance-responses, improve both technical and leadership competencies for malaria control, and gradually reduce overreliance on commodities while expanding multisectoral initiatives such as improved housing and environmental sanitation. They must also leverage increased funding from both domestic and international sources, and support pivotal research and development efforts locally. Effective vaccines and drugs, or other potentially transformative technologies such as genedrive modified mosquitoes, could further accelerate malaria control by complementing current tools. However, our underlying strategies remain insufficient and must be expanded to include more holistic and context-specific approaches critical to achieve and sustain effective malaria control.

## Introduction

Since its etiology was first described more than 100 years ago, malaria has become one of the world’s best-known infectious diseases. However, there were still more than 620,000 deaths and ~230 million cases worldwide in 2019, nearly all in sub-Saharan Africa [1]. A key question is why malaria control and elimination have proven so difficult in sub-Saharan Africa, while the disease has been either eliminated or greatly diminished elsewhere [2, 3].

Expert risk-benefit analyses suggest that malaria elimination would be both ethically and economically rewarding. Return-on-investment ratios are estimated at 40:1 globally and up to 60:1 in sub-Saharan Africa [4], yet there appears to be no consensus on whether eventual eradication is possible or whether it should even be pursued by the current generation [5]. A report by the Lancet Commission for Malaria Eradication argued that eradication by 2050, though ambitious, is achievable and necessary [6]. On the other hand, the World Health Organization (WHO) Strategic Advisory Group on Malaria Eradication (SAGme), while calling for greater investments for malaria control and R&D, avoided setting any specific target date for eradication—the group argued that even under the most optimistic scenarios, there would still be 11 million cases by 2050 [7]. Still, some individual countries and the African Union have set specified target dates for malaria elimination [8].

Relative to recorded history since 1900, unprecedented progress was made against malaria between 2000 and 2015 [9]. The development of simple but effective technologies, notably insecticide-treated nets (ITNs), in the 1990s helped renew international interest in malaria. These advances heralded major initiatives such as the 1998 formation of Roll Back Malaria and the 2000 seminal meeting by African heads of states and governments in Abuja, Nigeria [10], both of which laid the groundwork for the post-2000 malaria agenda. The establishment of major international funding agencies such as the Global Fund in 2002 [11], US President’s Malaria Initiative in 2005 [12], and the Bill & Melinda Gates Foundation in 2000 further unlocked significant increases in funding for malaria and attracted thousands of players to both R&D and implementation programs. WHO approved the first artemisinin combination therapies (ACTs), malaria rapid diagnostic tests, and long-lasting ITNs in 2000. In subsequent years, these commodities were scaled-up steadily via a series of policy decisions, culminating in the Global Technical Strategy for Malaria 2016–2030 (GTS) [13] and, most recently, the High Burden to High Impact country-led response [14]. The post-2000 advances in malaria diagnostics, treatments, and vector control have played a particularly crucial role by enabling historically high coverage and access rates across communities. The recent WHO recommendation for the use of the world’s first malaria vaccine RTS,S/AS01, known as Mosquirix, among children in moderate to high transmission areas is expected to advance these gains further [15]. The partially efficacious vaccine, if combined with existing control approaches, could be especially beneficial in areas with the greatest malaria burden [16].

Despite all these initiatives, the downward trend of malaria cases has plateaued since 2015, and in some cases, even reversed [1] as earlier predicted [17]. Africa now bears >95% of all malaria cases and deaths [1]. Part of this can be explained by expanding population size, but most malariologists typically blame the rise of resistance to insecticides or drugs [18]. However, these are only symptoms of broader strategic flaws and largely ignore key factors such as the potential of multisectoral approaches or the importance of effectively engaging communities and other stakeholders. Indeed, without multidisciplinary considerations, current scientific methods cannot adequately assess relevant associations between socio-economic variables and disease [19], a concept long championed to explain health outcomes in Europe in the 19th century [20].

As envisioned in both the GTS [13] and the SAGme report [7], malaria-endemic countries require combinations of interventions that are integrated and tailored to local contexts, as well as strong country ownership and leadership to accelerate progress through multisectoral approaches. Transformative technologies such as vaccines and gene-drive mosquitoes may one day quicken malaria control efforts and reduce costs by multiple orders of magnitude [21]. However, real progress currently requires more holistic strategies that effectively target the root causes of current and past failures. It requires an approach that does not ignore contextual complexities underpinning the delivery of malaria interventions and considers the needs of critical stakeholders including the communities, especially in rural and peri-urban areas.

This paper describes some of the most critical challenges in malaria control in Africa and highlights key opportunities for stakeholders to reflect and rethink malaria control and elimination. We review evidence from multiple sources and reflect on how countries might overcome these challenges and build sufficient momentum for a more realistic malaria control and elimination agenda in sub-Saharan Africa.

## Address weaknesses in health systems to maximize the effectiveness of malaria interventions

Formal and informal health systems are inherently complex and often require careful management of many non-linear relationships and components at play [22, 23]; nowhere is this more obvious than in the management of infectious diseases in low-income countries. Any health technologies tested under experimental settings must still be delivered and proven effective in real-life settings, a process with multiple quandaries including many that are only partially predictable. The COVID-19 pandemic further highlights the importance of strong systems and the inescapable interconnectedness of domains in ways not previously appreciated [24].

**Fig 1:**
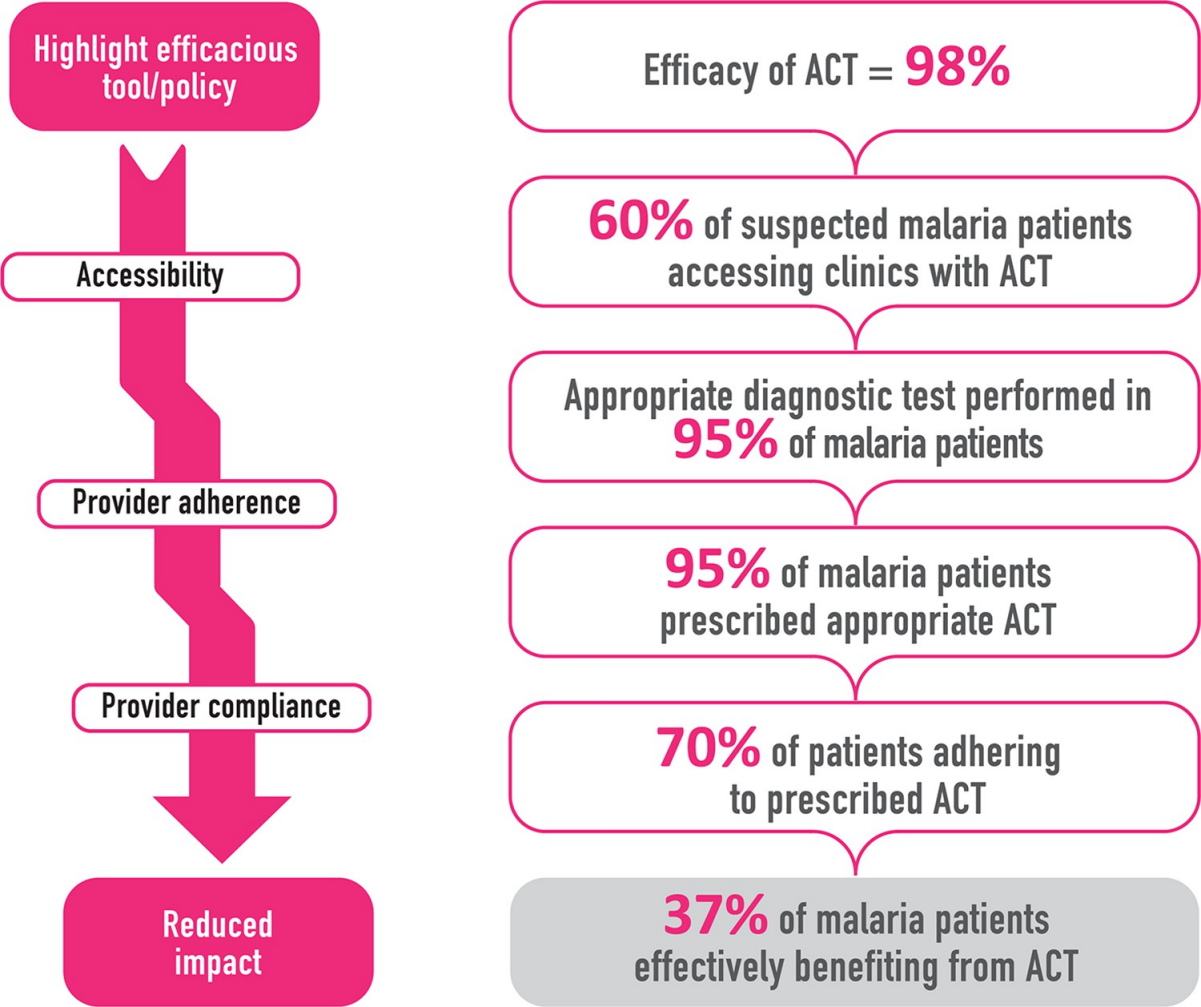
Illustration of how the effectiveness of key malaria control tools, in this case, Artemisinin Combination Treatments (ACTs), can decay due to multiple health system weaknesses in malaria-endemic countries (adapted from WHO/TDR implementation research toolkit [30]).

Health system weaknesses in malaria-endemic countries are a function of multiple factors that greatly limit the effectiveness of health commodities, thereby compromising both quality and timeliness of care. Countries particularly need good governance to ensure adequate resource acquisition and utilization, engage effectively with other relevant sectors, and ensure that all components of the health system function at equilibrium. In one study examining the implications of health system factors such as treatment-seeking, provider compliance, patient adherence to treatment and care, and quality of medication on treatment outcomes in 43 sub-Saharan African countries, the coverage of malaria case management ranged from as low as 8% up to 72% [25]. Indeed, even efficacious medicines such as ACTs, which have >95% cure rates [26, 27], may have as low as 20%–40% effectiveness due to health system weaknesses [28, 29]. Essential factors in this cascade may vary but broadly include poor accessibility, poor provider compliance with clinical protocols, suboptimal patient adherence due to socioeconomic and cultural factors, weak governance, and often supply chain constraints (Fig 1). These are compounded by inadequate knowledge in some communities for managing fevers, and care-seeking from unqualified providers and drug-stores due to the existence of pluralistic health care systems.

To address these challenges, implementation research capacity, which uses multidisciplinary approaches to identifying with care providers and program managers the challenges and bottlenecks related to the roll-out of health interventions should be prioritized by countries. Developing and testing effective strategies designed to overcome specific challenges, determining the best way to introduce innovations into the health system, and promoting their large-scale use and sustainability should also be incorporated at the country-level. Implementation research is characterized by the complex, iterative, systematic, multidisciplinary and contextual processes at multiple levels to identify and address implementation problems [31]. More often than not, this will include community-based participatory research in target areas [30].

Similarly, improved diagnostics are necessary to address challenges such as the rise of *HRP2/3* gene deletions [32] or poor validity of existing diagnostics in low-transmission settings [33]. Ultra-sensitive malaria rapid diagnostic tests have been proposed to improve detection under low transmission intensities or low parasite densities but risk biasing clinical management away from other febrile illnesses [34]. From a health systems perspective, the introduction of new diagnostic tools must therefore be part of a broader strategy for case finding and management, instead of purely focusing on the tools. One WHO technical consultation in 2018 noted the importance of assessing broader public health and clinical benefits for patients and communities but declined to recommend the highly sensitive point-of-care diagnostics for use in clinical settings [35].

These health systems concerns also extend to vector control interventions distributed and used in local households. For example, ITNs are most effective when accessible, appropriately used, and maintained or replaced regularly. While access is generally the key determinant of ITN use [36, 37], achieving high coverage and equitable access has been challenging without either expensive mass distribution campaigns [38] or a mix of deployment strategies [39]. Moreover, ITN use even among people with access requires consistent behavior change communication to maximize use [40].

While emphasing specific tools for malaria control and elimination (i.e., drugs, vaccines, insecticides, diagnostics) remains vital, endemic countries and their partners must ensure commensurate investments in their health system structures, most urgently for case management, to ensure as few deaths as possible.

## Improve data quality and data use to support malaria responses at national and subnational levels

The annual WHO malaria reports provide the most comprehensive overview of malaria trends globally. The reports are derived from voluntarily submitted records from more than 80 countries through systems of subnational cascading and summaries. Unfortunately, the lack of regularly collected high-quality data in many endemic countries, the WHO reports still rely primarily on mathematical models, with significant uncertainties around the actual estimates. When WHO recently made changes to the modeling methods, this required retrospective adjustments of early malaria estimates, evoking concerns over the apparent statistical inconsistencies [41]. Though the global agency regularly reports the actual methods of analysis and the statistical uncertainties around its estimates, these are rarely considered in day-to-day conversations or policy decisions, partly because of the detailed technical understanding required and the opacity of the methods to most stakeholders. A deeper analysis of these challenges points to weaknesses of in-country surveillance responses, notably poor-quality data, inadequate capacity to handle the data, failure to establish minimum essential data packages, and the wider disconnect between public health responses and data.

Many African countries currently use the DHIS2 software platform [42] to collect routine health care delivery data and manage their health information systems [43, 44]. However, there are concerns regarding the quality of the data collected and the inability of local program managers to analyze, interpret or use the data. Clinical data from private facilities, which are also left out should also be considered. Other concerns include poor integration of non-clinical data, particularly entomological data for malaria, which are not channeled via health facilities.

In addition to the first pillar (ensuring universal access to core interventions) and second pillar (accelerating efforts towards elimination), the third pillar of the GTS (2016–2030) suggested that malaria surveillance be transformed into a core intervention, alongside scale-up of effective vector control and case management [13]. However, most endemic countries remain too poorly resourced to establish effective surveillance-response systems with minimum essential data packages to detect changes and adjust public health responses [45, 46]. One consequence is that control strategies remain mostly unchanged and unlinked to local epidemiological transitions. One study reviewed national malaria strategic plans of 22 sub-Saharan African countries and examined targets for six core malaria indicators concerning reported population coverage [47]. It also analyzed implementation challenges and solutions proposed during in-country strategy discussions and whether the subsequent strategies integrated the lessons. Of the 135 verified targets, only four were achieved, and none of the countries had reached more than one-sixth of the targets [47]. Only four of 22 countries lowered relevant targets in their subsequent strategies. In fact, contrary to their own evidence, most countries either maintained or raised the targets and did not incorporate lessons from their own assessments.

The WHO’s High Burden High Impact response [14] requires that endemic countries have effective surveillance-response capabilities at both national and district levels. Unfortunately, countries targeted by the initiative still have far less capacity than countries approaching malaria elimination [45]. This paradox probably arose from previous strategies, which emphasized “shrinking the malaria map” from the periphery [48] and thus channeled significantly more resources per capita to low-burden countries in the periphery than to high-burden countries. Going forward, endemic countries must strengthen their surveillance-response practices and build relevant human resource capacity for these functions [49]. Countries should also collect data on the quality of care and implementation and include these in subsequent decisions to strengthen the sub-national stratification efforts.

Any minimum essential data packages should also consider relevant genomic data and address the associated challenges with computing infrastructure, genetic sequencing capabilities, and data sharing guidelines. The need for malaria molecular surveillance is increasingly evident in varying epidemiological settings to address multiple use cases [50], including enabling National Malaria Programs and partners to plan or deploy interventions proactively. Mathematical modeling may address some of these gaps by helping define the minimal essential data needs in space and time and focus areas, designing surveillance-response systems and making vital projections and resource allocation [51]. However, such strategies face even greater limitations in requisite skillsets in endemic countries.

Evidence from polio eradication programs suggests that high-quality data, including high-quality maps, combined with modeling are essential for targeting resources to the last hotspots and may offer lessons for malaria elimination, particularly with regard to health system needs, data requirements, essential technologies and partnerships, but more importantly for the design of integrated surveillance-responses [52, 53]. However, quantitative datasets from surveys, experiments, and genomic analyses often only inform what is happening, not why. For a complete picture, it is important to also incorporate qualitative datasets such as anthropological or human behavioral data to explain underlying drivers of vital empirical observations and unravel some of the religious and cultural nuances that influence perceptions about disease causation and healthcare-seeking behavior. It is equally vital to learn from other successful pathogen elimination campaigns, such as polio, guinea worm, and onchocerciasis, which have parallels in data quality and data use.

Countries must invest in building capacity for policymakers, program managers, frontline care providers, and their core district teams to interrogate, analyze, and use their data to plan and implement malaria control activities.

## Adopt more holistic and multisectoral approaches, instead of overreliance on imperfect commodities applied imperfectly

Early control strategies relied heavily on the basic biological understanding of malaria and how the natural environment influences human exposure to *Anopheles* mosquitoes. Despite limited economic opportunities, rudimentary technologies, and lower funding levels compared to today, countries that considered environmental management and improved housing as core interventions alongside other measures such as quininization made significant gains, and in some cases, remain malaria-free [3, 54].

However, once the highly effective insecticide DDT became the cornerstone of malaria control in the 1950s, further innovation for malaria control slowed. In recent years, widespread availability of ITNs and ACTs may have inadvertently reduced the appeal of more permanent but demanding measures such as environmental management and improved housing. These issues are compounded by the difficulty of using standard epidemiological study designs (e.g., cluster randomized control trials) to effectively measure the long-term impact of socioeconomic developments on disease [19]. For example, larval source management and improved housing clearly suppress vector densities and reduce biting risk, yet their epidemiological impact at scale is evident in only selected sites [55–57], and in some cases indemonstrable [58]. Similarly, housing is steadily improving in Africa [59], paid for mostly by individual household incomes, yet dominant epidemiological analyses of malaria trends only marginally examine the contributions of these trends.

Malaria control is now mostly dependent on commodities, namely drugs, diagnostics, medicines, and insecticides, which are imperfect and often deployed and used imperfectly. The commodities also must be replenished regularly, even as resistance spreads, manufacturing costs rise, and at-risk populations increase. This “commoditization of malaria control” also has caused significant declines in practical malaria expertise in endemic countries and instead incentivized fringe and disconnected players focusing on distribution and performance of the commodities. Major players regularly report short-term outputs, such as the number of treatment doses delivered, ITNs distributed, or houses sprayed, with only weak connections to epidemiological impact or effective delivery and use of these commodities. Indeed, there have been more than two billion ITNs [60] and one billion doses of child ACT formulations delivered, yet key malaria trends are stagnating. These decisions raise multiple questions, including whether the products meet actual quality thresholds or if there are certain imperfections. For example, despite manufacturer claims that ITNs last more than three years and 20 washes, recent studies suggest these nets last far shorter periods [61].

Moreover, while ITNs and indoor residual spraying (IRS) effectively tackle indoor-biting and indoor-resting mosquitoes, their effectiveness is limited in areas where significant biting happens outside homes or sleeping hours [62]. Another question is whether delivery of the commodities sufficiently covers all at-risk demographic groups, and commonly disenfranchised groups such as migrant and nomadic populations. Lastly, it demonstrates the importance of concurrent investments to build resilience in health systems and the environment and to build requisite human resource capacity to sustain gains catalyzed by current commodities and minimize the decay of effectiveness [28].

Endemic countries should realize that while ITNs, IRS, drugs, and diagnostics do indeed offer significant benefits against malaria in the short and medium-term [63], sustaining these gains requires a much more holistic approach. Greater focus on multisectoral initiatives, stakeholder including community engagement, one-health approaches (including considerations for essential agricultural practices and pesticide usage), more robust health systems linked to ecosystem approaches, and better behavior-change communication practices (involving community members and frontline health workers) will be essential to achieve real progress. In particular, environmental santitation must be incorporated as a key component of malaria control, for example by integrating ministries of environment, infrastructure, housing and other relevant sectors to keep communities mosquito-free. Depending on country-level administrative architecture, these other sectors and government agencies could play an important role of malaria prevention, thereby reducing case loads and allowing the ministries of health greater resources to improve case management.

These challenges are not unique to malaria, and must be addressed in the broader context of public health needs of individual countries. Decision-makers must accept that there may be certain functions best performed by sectors other than the health sector and that malaria programs should not be siloed. In many sub-Saharan African countries, the public health importance of malaria or the desire to achieve elimination has led to the creation of vertical control programs, in some cases disconnected from other disease programs or sectors. It is, however, important to maintain a reasonable level of integration in the wider context of sustainable development goals [64], particularly for improving peoples’ health and well-being.

## Increase both domestic and international funding for malaria control, research, and development

The estimated annual global budget for malaria control initiatives is ~$6.8 billion, yet only $3.3 billion was attained in 2020 [1]. A significant proportion of the overall financing for malaria control in Africa is currently from external sources, even in high-burden countries. Endemic countries’ contributions and direct investments rose steadily between 2001 and 2010 but have since stagnated at just under $1 billion annually. Only one third of the funds invested in malaria control over the last decade was from domestic sources, the remaining two thirds having been from external sources, principally the USA [1]. Given the economics of most malaria-endemic countries, it is not expected that domestic funding will match international financing soon, but there may be additional opportunities to attract internal funds for control and elimination efforts.

It can be difficult to track malaria-related expenditures, especially since domestic funds may be tied to multiple recurrent costs or salaries, and because there may be many indirect payments by the multiple agencies involved. Besides, where the private sector plays a significant role in health care, such data may not be readily reported in standard government portals. One study analyzed domestic malaria spending by source in 106 countries from 2000 to 2016 [65], considering data for out-of-pocket payments, private insurance prepayments, costs for treatment, patient care and direct drug purchases. The study also estimated malaria-related government spending within and beyond National Malaria Programs. The results estimated that since 2000, out-of-pocket spending increased by 3.8% annually, to 13% of total domestic financing for malaria by 2016, and that endemic country governments had spent $1.2 billion the same year [65]. It is expected that the countries will indeed increase their investments in the future, with some countries already taking the lead. In Ghana, where malaria elimination is estimated to require $1 billion by 2029, government expenditure on malaria control is expanding, though this is still below 25% of total funding [66].

Limited local investment may reduce in-country responsibility for malaria control and the premium placed on monitoring progress of this infectious disease. These issues particularly affect low-income households by constraining their incomes and challenging other competing priorities, especially where the opportunity costs associated with malaria control are high. The economic burden of health care is well-documented and can be massive [67] or even catastrophic for low-income households [68]. Without removing financial barriers for these households, basic health-seeking behaviors and treatment will likely be deprioritized in favor of alternative medicines or other household needs such as food. In Tanzania, researchers asked household heads whether they knew that unscreened windows and eave gaps in their houses were a risk factor for malaria [69]. They found that community members were aware of these risks and desired to make improvements, but they were constrained by competing priorities [69]. In many communities, healthcare-seeking behaviors and investments for health are influenced significantly by household-level decision-making processes, which have economic, cultural, and social determinants and differ across settings [49]. Thus, it may be beneficial to have household-centered approaches and to consider these factors when designing universal health coverage packages.

These difficulties in funding, coupled with the desire by international donors to track specific malaria program indicators, have further entrenched the vertical structures of malaria control, which are sometimes siloed from other functions of the health sector. The vertical approach to malaria control misses significant multisectoral opportunities to catalyze or sustain gains [70] and unlock additional resources [49]. For example, there are certain aspects of vector control, such as larval source management and improved housing, that are best managed by government ministries beyond health. Moreover, collaborating with sectors such as tourism and finance could unlock additional financing necessary for malaria control. Where feasible, appropriate legislation could further improve compliance, protect vulnerable people, and guarantee long-term domestic financing for malaria.

Similarly, despite its growth, the private sector market, remains neglected yet could support local supply and distribution of essential commodities such as ITNs or medicines [71, 72]. Estimates from Ghana suggest that the private sector market could free nearly 40% of investments currently incurred by ITN distribution systems that do not consider individual household preferences and willingness to pay [73]. Greater involvement of the private sector also may generate additional positive externalities, such as local manufacturing of essential tools such as ITNs [74]. Unfortunately today, even the most-affected African countries—such as Nigeria, Democratic Republic of Congo, Mozambique, Uganda, Niger, Burkina Faso, Ghana, and Cameroon, which together constitute nearly 70% of the global malaria burden—still regularly import bed nets, insecticides, and medicines to protect their citizens. Local initiatives such as mosquito net manufacturing and greater private sector involvement could increase ownership, reduce overall costs and expand access to interventions.

Beyond direct investments for malaria control, there is a need to accelerate investments for R&D, especially on potentially transformative tools such as vaccines and gene drive mosquitoes. Besides the many technical challenges of developing transformative technologies, particularly vaccines [75], the innovation pathway for malaria remains poorly funded and takes far longer than other diseases. It is worth noting that malaria etiology was first described in 1880, yet no viable vaccine has achieved full approval [76]. In rethinking malaria, African Governments in particular must stop the rhetoric and increase investments in control and R&D for a disease that remains a leading killer on the continent. Given the reality of significant funding gaps, the growth of indigenous funding should not be interpreted as a reason to reduce international funding.

## Recognize that the epidemiology of malaria in Africa is more challenging than elsewhere; and is compounded by poverty and multiple biological threats, notably resistance to insecticides and drugs

Compared to sub-Saharan Africa, malaria control has advanced much faster in other geographies. The epidemiology of malaria in sub-Saharan Africa is particularly more challenging than elsewhere. The high levels of poverty in Africa has historically hindered progress against infectious diseases, and must be addressed gradually and comprehesnsively for long-term progress to be realized. While this is typically beyond the scope of health ministries, countries must broadly consider malaria control in the context of sustainable development goals and additional justification for eventual elimination.

Regarding the prevailing epidemiology, dominant Afro-tropical malaria vectors, *Anopheles gambiae*, *Anopheles funestus*, and *Anopheles colluzzi*, have the highest propensities to bite humans over other hosts [77] and are among the most competent malaria vectors worldwide. One study examined the stability of malaria in relation to multiple factors and derived an index for the epidemiological contribution of dominant malaria vectors in different regions of the world [78]. Key factors included in this analysis were: i) the human blood index (i.e., the proportion of blood meals taken from humans instead of other vertebrates), ii) the daily survival probabilities of individual mosquitoes, iii) the duration of the year when malaria transmission is possible, and iv) the incubation period of malaria parasites in mosquitoes. The study concluded that the superior potential of many tropical *Anopheles* means that control efforts in sub-Saharan Africa are far more complex than in other formerly endemic countries [78]. A recent simplification of these stability maps, showing just human blood index values, also depicts the region as most amenable to malaria transmission given the extremely high degree to which local *Anopheles* vectors prefer humans [79].

The spread of insecticide resistance in vector populations has further complicated the situation, meaning that formerly impactful interventions such as ITNs and IRS now have less impact. Similarly, formerly effective medicines such as chloroquine [80] and sulfadoxine pyrimethamine are no longer suitable, and there are signs of artemisinin resistance now arising *de novo* in Africa [81]. Additionally, although people’s knowledge about malaria may have improved across endemic countries, the changing epidemiology means that this knowledge is due for a gradual update. For example, farming communities should be aware of risks associated with their practices and how wanton application of pesticides may contribute to insecticide resistance and consequently, poor performance of ITNs and IRS [82]. As highlighted in the most recent WHO world malaria report [1], there are several other biological threats converging over Africa, notably: a) emerging resistance to key antimalarial medicines, b) reduced performance of mRDTs, c) the recent invasion of the Asian mosquito, *Anopheles stephensi* mosquito in the horn of Africa, d) major epidemics, such as Ebola and COOVID19, and e) wars and coflicts.

Additional factors contributing to greater malaria transmissibility in Africa include human behaviors and occupational exposures. Despite the success of core interventions such as ITNs, residual malaria transmission is in many areas perpetuated by human behaviors or activities that overlap with malaria vector biting exposures beyond actual bed-times [83]. Outdoor biting risk is often discussed as a byproduct of mosquitoes changing their behaviors in response to ITNs and IRS, but it also is a function of human behaviors and practices [84]. In some communities, migrant workers, forest workers, fishing communities, or nighttime staff such as security play an important role in residual malaria transmission. Poor knowledge and exposures associated with these practices may perpetuate malaria risk. The mostly rural population in Africa also has lower access to behavior-modifying factors such as electricity, or mosquito-proof housing and thus spending extended periods outdoors.

Malaria control initiatives in Africa should be both comprehensive and context-specific to address the region’s unique epidemiological challenges. With recent progress starting in 2000, there also is an increasing degree of within-country variation [85], necessitating subnational stratification to better set priorities and effectively allocate resources to be more impactful in decentralized health systems [86].

## Improve technical capacity and leadership for public health practice and research in malaria-endemic countries

Effective malaria control and elimination require well-trained and experienced practitioners and leaders at all levels [49]. It is the human resource that brings together the various aspects necessary to address malaria quandaries such as those addressed above. Beyond financing and implementation policies, technical expertise is needed to adopt the best practices to suit local contexts and manage effective surveillance-response programs. In addition, countries also require implementation research capacity to identify and address arising challenges readily. Tackling these issues requires that capacity building is addressed not as a short-term endeavor but as a long-term program focusing on people’s careers, institutional ecosystems, and long-term mentorship. It also requires a comprehensive view that considers the scientific components of malaria control and the public health administration and related services.

After decades of inaction, the 2000 Abuja Declaration by African Heads of State and Government, following other events such as the founding of the “Roll Back Malaria Partnership to End Malaria” (then Roll Back Malaria), catalyzed renewed interest that heralded malaria declines in the post-2000 era. This was a rare show of political leadership and commitment that has not been evident more recently. Instead, global efforts for malaria control are today led mainly by international experts and international agencies, which direct the in-country experts. Going forward, it will be important that African countries take control and leadership of their malaria programs instead of the leaving most of the responsibility to the international community as is currently the case.

Limitations in capacity are widespread, although specific disciplines such as social sciences, genomics, data sciences, and research to policy translation appear to be much more affected [49]. Though Africa is the most affected by malaria, influential research about the disease is still overwhelmingly led by non-African researchers and institutions. While these gaps are gradually closing, thanks to improved global health collaborations, African researchers are still far less represented in leadership and agenda-setting. One 2019 study investigated how international collaborations affect the representation of local authors in health research conducted in Africa [87]. Nearly 70% of the publications had evidence of international collaborations, most of these with North American and European scientists. However, more importantly, only 41% of all authors and only 23% of first-name authors were from the respective target countries, and 14% of all papers had no local authors [87]. Addressing these gaps will enable greater responsiveness to Africa’s needs and better opportunities to address the identified challenges effectively. The importance of capacity building for malaria control also is core to the broader health system initiatives and multisectoral initiatives. The malaria elimination agenda will require skilled personnel with broad understanding across disciplines as well as people with strong focus on particular fields to advance the testing and implementation of new interventions being developed. The need for trained staff at both national and district levels and last-mile operatives will be essential at all levels of transmission, even though current evidence suggests that countries approaching elimination are better staffed than high-burden countries [45].

As sub-Saharan Africa rethinks the malaria control agenda, countries must expand high-quality training from basic to tertiary levels. Such training will impart skills relevant for addressing the challenges identified here, the effective deployment of interventions across epidemiological settings, and the development and evaluation of new interventions. Given the socio-economic basis of infectious diseases, countries should expand this research capacity to include methods for assessing the associations between socio-economic variables and malaria and to also demonstrate the health impact of these types of intervention.

The training should be expanded to different people involved in the supply chain for medical supplies, including distributors, vendors, and regulators. A detailed mechanism for building relevant capacity can be found in the paper in this “Rethinking Malaria” series by Mwenesi and Mbogo [49].

## Other challenges to address

The issues raised above are only key examples of the many quandaries of malaria control and elimination programs in Africa. Other challenges include: i) political instability, conflicts, and displacements in some countries, which may compromise efforts to strengthen health systems, conduct relevant research or develop practical tools; ii) disconnected health care systems through ill-defined pluralism and too many partners often working without unified strategies; iii) varied cultural beliefs and unproven traditional practices about malaria and its management, which may reduce appropriate health-seeking and compromise effectiveness of case management; iv) other disease epidemics such as COVID-19 and Ebola, which may disrupt implementation of malaria control activities and reduce political commitments on malaria [88]; v) the looming threat of climate change, which could further expand the geographic range of transmission, increase population vulnerabilities, and reverse previous gains [89]; vi) replacement vectors or invasive vector species such as *Anopheles stephensi*, now established in the horn of Africa, and their potential to spread [90]; and vii) inadequate communication leading to insufficient community knowledge and participation, viii) some human behaviors and practices which reduce compliance to interventions and ix) the steadily increasing populations in endemic countries leading to greater demand for malaria control.

While these additional challenges were not discussed in detail in this paper, they too must be monitored carefully to enable effective implementation of malaria control and elimination programs that are appropriately adapted to local conditions.

## Conclusion

Rethinking malaria control and elimination strategies is imperative. Holistic and systemic approaches that include communities and households to effectively stop transmission and deaths are needed. The exceptionally challenging epidemiology of malaria in Africa requires context-specific initiatives tailored to national and subnational targets. In addition, endemic countries should address the weaknesses in their health systems, improve the quality and use of data for surveillance-responses, improve technical and leadership competencies for malaria control and reduce overreliance on commodities while expanding multisectoral initiatives. The countries should also invest more in malaria control as well as on key research and development agenda, including on potentially transformative technologies such as vaccines and gene drives. Lastly, to complement these efforts, countries should build requisite resilience and capacity to broadly enhance infectious disease control.

## Supporting information

S1 Text "Rethinking malaria in the context of COVID–19," a global engagement organized by Harvard University. (DOCX)
